# Mindfulness meditation for pulmonary hypertension program development: Acceptability and feasibility of technology-assisted complementary health intervention for symptom self-management

**DOI:** 10.1016/j.hrtlng.2026.102759

**Published:** 2026-03-12

**Authors:** Tania T. Von Visger, Yu-Ping Chang

**Affiliations:** a Assistant Professor, The Medical University of South Carolina, College of Nursing, Charleston, SC, USA; b Associate Dean for Research and Scholarship, Associate Professor, School of Nursing, The State University of New York, University at Buffalo, Buffalo, NY, USA

**Keywords:** Mindfulness, Meditation, Pulmonary hypertension (PH), Intervention fidelity monitoring (IFM), Complementary health approach (CHA), Mindfulness mobile app, Acceptability, Feasibility

## Abstract

**Background:**

Adults with pulmonary hypertension (PH) live with a high symptom burden, psychological distress, and poor quality of life. Mindfulness-based interventions delivered in person or virtually have shown clinical benefits for chronic conditions and PH. Pilot testing of the Mindfulness Meditation for Pulmonary Hypertension (MMPH), an 8-week, web-based, multimodal program, shows improvement in depression, symptoms, and quality of life.

**Objectives:**

To evaluate the MMPH program’s acceptability and feasibility quantitatively and qualitatively from MMPH participants.

**Methods:**

Descriptive statistics were used to report adherence to and completion of each component of MMPH (Zoom, video, and app use) among 15 participants who received the intervention in 2022–2023. Qualitative thematic analysis was conducted on their daily reflection journals, downloaded from the MMPH app.

**Results:**

Fifteen participants attended 28 out of 30 Zoom sessions (93%). Twelve of 15 (80%) participants accessed the video-viewing activity at least 50% of the time (3 of 6 videos). Eight out of 15 participants (53%) used the App during the intervention period, and 6 out of 15 participants (40%) used the App for >2 weeks. The content analysis reveals four most salient themes: Perceived Benefits of Meditation, Daily Life Practice and Activity, Awareness and Acceptance, and PH Symptom Improvement. The System Usability Scale for the MMPH program was 81.47 (±10.21), indicating moderate acceptability.

**Conclusions:**

Assessing the feasibility and acceptability of a complex behavioral health intervention, such as the MMPH program, is critical to ensuring consistent intervention fidelity. Comprehensive approaches are essential to guide necessary program modifications for future real-world implementation science testing.

## Introduction

### Pulmonary hypertension (PH)

Pulmonary hypertension (PH) is a debilitating and progressive cardiopulmonary (CP) condition due to multiple genetic and pathophysiologic etiologies that ultimately result in an abnormal elevation in pulmonary arterial pressure. Given the diverse etiologies of PH, diagnostic confirmation is often complex and delayed, leading to the post-ponement of timely treatment for patients with suspected PH. The complexity of this condition led to the development of a standardized diagnostic confirmation procedure involving right-sided cardiac catheterization, which indicates an elevation in mean arterial pulmonary pressure (>20 mmHg).^1^

PH is a complex life-limiting cardiopulmonary condition, specifically among older adults and non-white populations.^2^ The overall prevalence of PH in the US is difficult to pinpoint due to multiple etiologies that can lead to this debilitating and possibly end-stage PH. PH is categorized into five World Health Organization (WHO) groups based on underlying causes: Group 1 PH (labeled as pulmonary arterial hypertension (PAH)), Group 2 PH (due to left heart disease), Group 3 PH (due to lung disease), Group 4 PH (due to chronic blood clots in the lungs), and Group 5 PH (due to other causes).^1^

Estimates for the number of people living with each PH group in the US vary by group, with more precise epidemiological data for some groups than others. Group 1 PAH is rare, while Groups 2 and 3 are much more common due to underlying cardiac and pulmonary diseases; data for Groups 4 and 5 are more limited. An estimated 1.5 % of adults with severe COPD will develop Group 3 PH, and about 60–70 % of adults with severe heart failure develop Group 2 PH Severe COPD is classified as GOLD Grade 3, which is based primarily on spirometry results according to the Global Initiative for Chronic Obstructive Lung Disease (GOLD) criteria.^3^ PH due to heart and lung diseases may be underdiagnosed, resulting in an underestimate of PH. Considering all these factors, an estimate of 339,000 to >1 million US adults living with PH with variable medical treatments among the five PH Groups.^4–9^

The anatomic and physiologic abnormalities lead to high symptom burden^10^ and psychological distress symptoms (anxiety and depression),^11^ contributing to difficulty in symptom self-management.^12^ At the same time, there has been enormous progress in pharmacological therapeutic approaches to managing this condition during the past 20 years; however, patients with PH continue to live with high symptom burdens and poor quality of life, challenging self-management of this chronic symptom. Complementary health approaches that integrate mindfulness practices to augment PH daily self-management have shown therapeutic benefits, including reduced symptom burden and improved quality of life.

### Mindfulness-Based interventions (MBIs)

Since the development of the eight 2.5-hour weekly *Mindfulness-Based Stress Reduction (MBSR)* program, created by Dr. Kabat-Zinn, numerous modifications have been made to the program to meet the needs of adults with various chronic conditions. Chronic-disease-specific MBSR programs have been tested in the past decades, showing mental health benefits in chronic depression,^13^ multiple sclerosis,^14^ and breast cancer.^15–17^ Advancements in technology, improvements in internet connectivity, and the COVID-19 pandemic have contributed to a greater interest in developing and designing technology-assisted mindfulness interventions, which address self-guided mental health needs in the safety and privacy of the home environment. Mindfulness-based interventions (MBIs) are behavioral health interventions that incorporate mindfulness practices to facilitate positive behavioral health outcomes. MBIs have demonstrated positive clinical outcomes, such as depression and anxiety reduction, and exercise capacity in patients with chronic obstructive pulmonary disease (COPD).^18–22^ Among other chronic disease populations, MBIs have been shown to improve sleep,^23^ enhance exercise capacity,^24^ and reduce depression and anxiety.^25^ Specific to PH populations, the application of MBIs remains limited, likely due to the challenges of MBI delivery for this debilitating patient population.

MBIs have been tested among many populations with chronic health conditions, demonstrating positive psychological and quality-of-life outcomes. MBIs can be delivered through various methods, including in-person, web-based, or in combination, each with its own advantages and disadvantages. Variability in delivery methods, driven by the need to modify the original MBSR program for specific patient populations, leads to differences in clinical outcomes across these populations. Despite this variability, as a complementary health approach, MBI has shown a positive impact in reducing psychological distress, such as depression and anxiety, without added side effects compared to pharmacological interventions. Web-based MBIs alleviate symptom burden and depressive symptoms for people with physical health conditions, particularly when interventions are tailored for specific symptoms.^26^ Specific research studies conducted on pulmonary patients have virtually delivered mindfulness interventions (video viewing, Zoom connection, and Mindfulness App) to COPD patients, showing that MBI has comparable efficacy to in-person interventions if patients adhere to the intervention.^27^ Although general MBIs are effective across many chronic conditions, they may be less suitable for populations with condition-specific physiological and psychological stressors. PH is associated with exertional dyspnea, fatigue, and activity intolerance, as well as high levels of illness-related anxiety and uncertainty. Standard mindfulness practices, particularly breath-focused or prolonged seated meditation, may exacerbate distress in individuals with PH by increasing symptom awareness. Therefore, an MBI tailored to PH allows adaptation of core practices to accommodate these constraints, improving safety, acceptability, and engagement while more directly targeting PH-specific mechanisms of distress and enhancing the potential for meaningful clinical benefit.

### Mindfulness-Based interventions (MBIs) in PH

There have been few MBI studies specific to the PH population. The largest randomized controlled trial of in-person MBSR in PH Group 1 (PAH) was conducted in the UK, yielding inconclusive results due to limited patient enrollment.^28^ Several pilot studies have been conducted in PH using a multicomponent CHA intervention delivered in an in-person format, Urban Zen Integrative Therapy (UZIT), which was associated with reductions in PH-related symptoms such as pain, dyspnea, anxiety, and fatigue, and improvements in PH-related quality of life.^29^ The intervention was well accepted, as indicated by a qualitative thematic analysis of the study participants.^30^ Of critical importance, the UZIT research study demonstrated high internal validity, as evidenced by careful measurement of the five crucial criteria for intervention fidelity.^31^ Because UZIT was designed for in-person delivery, its implementation became prohibitive during the COVID-19 pandemic, prompting its adaptation for virtual delivery. The Mindfulness Meditation for Pulmonary Hypertension (MMPH) intervention was designed for Zoom, videos, and app delivery while retaining the same practice routines as from UZIT. The MMPH program was pilot-tested and has shown preliminary efficacy in reducing depressive symptoms.^32^ Both the UZIT and MMPH programs were designed and tested to address the psychological distress and symptom self-management needs of community-dwelling patients with PH, where MBI availability and access are limited for people with PH pH.

### Acceptability and feasibility of MBIs

MBIs, delivered in either in-person or virtual format, must include Acceptability and Feasibility metrics that document participants’ level of engagement and dose receipt. These metrics not only substantiate adherence levels but also identify areas that require modification to achieve optimal program design. Complementary health approaches, such as mindfulness, have reduced burdensome symptoms for patients with COPD conditions^22,33^ and PH patients^29^; however, the consistency of how and how much the intervention is delivered *(intervention fidelity measurement)* is often insufficiently reported.^34^ As a behavioral health intervention, an MBI frequently includes multiple components aimed at acquiring knowledge and demonstrating the learned knowledge and practices. To appreciate the impact of the intervention on health outcomes, the researcher must assess intervention fidelity along with the clinical outcomes of interest. The behavioral health intervention must demonstrate adequate intervention fidelity and be well-designed with adequate measurement of participants’ “buy-in” or *Acceptance*. More importantly, the intervention should also demonstrate *Feasibility* from the participants’, interventionists’, and systems’ perspectives. This comprehensive view of intervention feasibility is critically important in a research program focused beyond pilot testing of an intervention (Table 1).

While the conceptualization of feasibility within intervention development involves multiple iterative processes,^35^ asserting that an intervention is feasible raises the question: “Can this be done?” While this basic definition of feasibility from participants’ and interventionists’ perspectives is an excellent starting point, we must consider the feasibility framework from the system’s perspective to sustain and build the evidence appropriate for large-scale benefits. Therefore, assessing the acceptability and feasibility of a behavioral health intervention may require considering multiple variables along the trajectories of research phases. Researchers must identify appropriate and relevant variables specific to the type of behavioral health intervention and the phase of research inquiries. Since the MMPH was pilot-tested in PH to integrate it into the existing healthcare system, it is necessary to ascertain its feasibility and acceptability for future sustainability.

When considering what to measure and how to assess the acceptability and feasibility of a behavioral health intervention at each stage of research, researchers should be mindful of the broader perspective of the entire study process. At the fundamental level, assessing the preliminary impact of an intervention requires that the treatment be delivered with a consistent dose and method, which is the most reported aspect of intervention fidelity measurements. The intervention’s consistent dose and delivery enhance the internal validity of the research, which, in turn, extends to the intervention’s generalizability. Therefore, delivery consistency is the most commonly reported aspect of behavioral health intervention studies, while the other four criteria are omitted. To strengthen the quality of behavioral health intervention research, the NIH Behavior Change Consortium (BCC) developed intervention fidelity guidelines that encompass the entire research study conducted.^36^ The guidelines include a more comprehensive assessment of the study intervention across the research conduct: *study design, provider training, treatment delivery, treatment receipt, and enactment of treatment skills*.^36^ Given the pilot test of the MMPH intervention for PH, we focus on treatment delivery and receipt. Through this process, behavioral health complementary interventions can be rigorously tested with high fidelity and generalizability to the target population, yielding the greatest benefits for mental health and outcomes.

## Methods

### Study aims and purpose

This research report aims to describe the Acceptability and Feasibility of the MMPH intervention from the participants’ perspective. While intervention acceptability can be considered from multiple conceptual frameworks,^37^ it is operationalized as measurable factors, including dropout rates, the degree of MMPH intervention delivery and receipt, and satisfaction measures. Feasibility can be broadly defined as the capability for recruitment, data collection procedures, design procedures, practicality, and integration with existing systems.^35^ In this pilot study, we adopted a more focused definition of the MMPH participants’ capability to perform the intervention tasks as outlined in the research protocol. Feasibility, therefore, is measured from the end user’s perspective as a foundational understanding of this multicomponent behavioral health intervention. It is essential to report on the fidelity metrics, particularly when research involves: 1) designing and testing a complementary intervention, 2) behavioral health modification, 3) multiple component interventions, 4) rare disease conditions of PH, and 5) chronic progressive conditions. The indicators of acceptability and feasibility used in this pilot study are summarized in Table 1.

The preliminary efficacy results from the MMPH Intervention testing, conducted in a pilot-randomized, delayed intervention study design involving 15 participants with PH, demonstrated statistically significant improvements in depressive symptoms, along with enhanced mindfulness and health-related quality of life.^32^ This current study report describes the *Acceptability* and *Feasibility* metrics of the MMPH intervention from the perspectives of participants who completed this study from August 2022 to November 2022.

### Ethical considerations

All participants in this study provided informed consent, as approved by the Institutional Review Board (IRB) for human research subjects (IRB number: 00,005,746).

### Sample and recruitment

We began participant recruitment and enrollment after receiving Institutional Review Board approval. Sixteen community-dwelling adults with PH were invited and agreed to participate in the study by early August 2022. Given the exploratory nature of this feasibility study, our data analysis focused on identifying preliminary signals of the MMPH intervention’s efficacy rather than establishing statistically significant differences between groups. Therefore, the sample size was determined pragmatically, as advocated by Kraemer and colleagues,^38^ based on assessments of dropout rates, recruitment, and retention. Inclusion criteria were: (1) adults (>18 years); (2) PH diagnosis confirmation by PH physicians; (3) willingness to participate in the mindfulness-practice program; (4) ability to ambulate independently; (5) English-speaking; and (6) having access to a mobile phone. Exclusion criteria were: (1) known pregnancy; (2) having psychiatric conditions requiring hospitalization within the past year; (3) being a current practitioner of mind-body practices; (4) current user of mindfulness apps; and (5) being deaf or hard of hearing.^32^ We approached potential eligible patients through referrals from their PH physicians and face-to-face recruitment during the 2022 Pulmonary Hypertension Conference Research Room. As a pilot, randomized delay-treatment controlled trial, Group 1 participants (*n* = 9) received an 8-week MMPH intervention in the first half of Study Week #1 through #8 (August-September 2022), and Group 2 participants (*n* = 7) received the intervention in the second half of Study Week #9 through #16 (October-November 2022).

### MMPH program intervention

MMPH is an adapted version of the UZIT intervention, utilizing available technology-assisted delivery of the MBI content prescribed in the UZIT, which has shown preliminary efficacy in improving mindfulness, depressive symptoms, and health-related quality of life.^32^ MMPH content was modified in collaboration with PH adults who previously participated in the UZIT research study (Table 2). During our meeting, we asked them to envision how similar content would be delivered virtually, given their current experiences with health-related technology (e.g., Apple Watch). Based on feedback from UZIT participants, we modified the MMPH intervention to include eight weekly 60–90-minute sessions, encompassing three delivery methods: Zoom meetings (Weeks 1 and 4), Video Viewing, and daily use of the MMPH App. We will report the feasibility and acceptability metrics of each MMPH intervention delivery method (Zoom, videos, and app), specific to 1) content descriptions, 2) delivery protocol, and 3) participants’ adherence.

The MMPH intervention included three main components: 1) Panopto Video Viewing, 2) Zoom meetings participation, and 3) MMPH mobile App use.

### MMPH panopto videos

The research protocol requires participants to view the 60-minute pre-recorded videos at the beginning of study weeks 2, 3, 5, 6, 7, and 8. Each weekly content covers mindfulness concepts and practices in a progressive format that begins with an introduction to mindfulness, followed by symptom management related to PH, stress management related to PH, mindful communication, healthy behaviors related to PH, community connections associated with PH, and fostering ongoing mindfulness practice (Table 2). The content structure of each recording is presented in a consistent sequence: 15 min of weekly content explanations, 15 min of gentle body-movement exercises, 15 min of body-awareness meditation, and 10 min of body positioning for comfort. We sent the access URL link to each week’s video at the beginning of the study week and instructed participants to view and practice mindfulness at least once during the week. Although each recording is released at the start of the week, participants can access all available videos until the end of the MMPH study participation period.

### MMPH zoom meetings

The research protocol requires participants to attend two 90-minute Group Zoom Sessions, one in week 1 and one in week 4. The time and date of these Zoom meetings were scheduled at a convenient time for the group facilitator, the group co-facilitator, and participants (either Group 1 or Group 2). If participants are unable to attend the Zoom sessions, they can reschedule the meeting with the group facilitator for a later date and time within the same week. Participants received their Zoom sessions with a consistent team of facilitator-co-facilitators. The group facilitator is an experienced mindfulness instructor, certified in the MBSR. She is also a registered nurse with 35 years of clinical experience and 20 years as a clinical nurse specialist caring for adults with PH across clinic, hospital, and critical care settings. She self-identified as Asian American. The co-facilitator is a certified yoga teacher with 15 years of experience and a mindfulness teacher with 10 years of experience working with community-dwelling adults with chronic conditions. She self-identified as African American.

### MMPH appdaily use

The research protocol requires participants to access the MMPH App daily to facilitate their mindfulness practice for at least 20 min/ day from the first day of Week #1 through the last day of Week #8. The App houses 15 audio recordings, ranging from 5 to 20 min in length (Table 2). These recordings are organized weekly according to the recommendations of expert mindfulness practitioners with prior experience in-person UZIT delivery. The audio content encompasses four categories of mindfulness practices: mindful breathing, gentle body movement, body-awareness meditation, and body position for comfort. Weekly content categories in the MMPH App align with the weekly video content and are designed to augment and facilitate participants’ daily practice. In addition to the expert recommendation, these audio recordings are organized with consideration of the feedback from PH patients in the prior UZIT study. We tailored body movements and positions, considering their progression and learning expectations, as well as their mindfulness experience, while remaining true to the original MBSR program content. To facilitate their learning and engagement, earlier practices include shorter modules (5 min), whereas later ones include longer modules (20 min). Within the App, participants can provide daily check-ins on symptom severity and enter weekly reflections to document their mindfulness journey.

## Measurements

In addition to assessing the indicators of acceptability and feasibility summarized in Table 1, we also used the System Usability Scale (SUS) to objectively evaluate the overall acceptability of the MMPH intervention. The original SUS was created to assess the acceptability of technology use in the general population.^39^ The tool was modified and used with suitable reliability in the PH population for the UZIT intervention.^31^ The SUS-M contains 13 Likert-type questions, some of which are reverse-scored, measuring the ease of use of the MMPH program. The SUS provides a global usability score ranging from 0 to 100, with 68 representing the average acceptable usability benchmark. Scores above this threshold indicate better-than-average usability, while scores below suggest usability concerns. Interpretation guidelines classify scores ≥85 as ‘Excellent,’ reflecting highly intuitive systems suitable for broad clinical implementation. Scores between 70 and 84 are considered ‘Good,’ suggesting minor refinements may enhance user experience. Scores from 50 to 69 indicate ‘Marginal’ usability, requiring significant improvements before adoption, and scores below 50 reflect poor usability and the need for redesign. Participants were also invited to document their reflections in the app regarding their experiences with the app content.

## Analysis

Descriptive statistics were used to report the mean and standard deviation of the dataset. We use SPSS v. 22 statistics software to manage and analyze data. We employed a qualitative content analysis^40^ to describe the salient themes in the downloaded reflections collected over the 8 weeks participants used the MMPH app. An experienced qualitative researcher reviewed the downloaded daily reflection content from the beginning to the end of the intervention (Weeks 1 through 8) and developed codes and corresponding descriptions. These codes and their definitions were reviewed and confirmed by the second qualitative researcher (TV) during regular meetings. After the final themes were compiled, the third qualitative researcher (YC) reviewed and resolved any inconsistencies. Any unclear points or disagreements about the finding summary were discussed among the three researchers, resulting in the final thematic summary.

## Results

### Quantitative results

#### Weekly MMPH sessions attendance

One out of 16 participants who provided informed consent declined to participate in the study because they could not commit to an 8-week-long intervention. Enrollees were 15 PH adults with a mean age of 51.73 (±11.92) and 93.3 % female (Table 3). The mean System Usability Score was 81.47 (±10.21), reflecting moderate acceptability of the MMPH program.

Table 4 displays the total time spent by the remaining participants in each weekly MMPH session. This result summary pertains to the combined results of video viewing among 15 participants. Twelve out of 15 (80 %) participants accessed the video viewing activity at least 50 % of the time (3 out of 6 videos). The number of participants who watched the video each week ranged from 8 to 10. Among those who accessed and watched these videos, the percentage of content viewed varied, likely due to multiple viewing sessions (indicative of a total viewing time exceeding 100 %). Table 4 shows that some videos were viewed multiple times by participants (ranging from 1 to 6). The total time participants spent reviewing weekly video content ranged from 279.6 to 593.9 min. The average of video content viewing in Weeks #2, #3, #5, #6, #7, and #8 was 101.48 %, 103.38 %, 49.88 %, 55.69 %, 86.53 %, and 50.03 %, respectively, indicating a gradual decline in content viewing and practice (the % above 100 is indicative of multiple times participants accessed the videos). Among the 14 participants who consistently watched the videos, 12 (85.71 %) viewed them more than once. The frequency of video views and downloads declined over the course of the study (26, 25, 15, 13, 13, and 11, respectively). Overall, participant engagement with the videos decreased from weeks 5 through 8.

#### MMPH app engagement

**Fig. 1.**
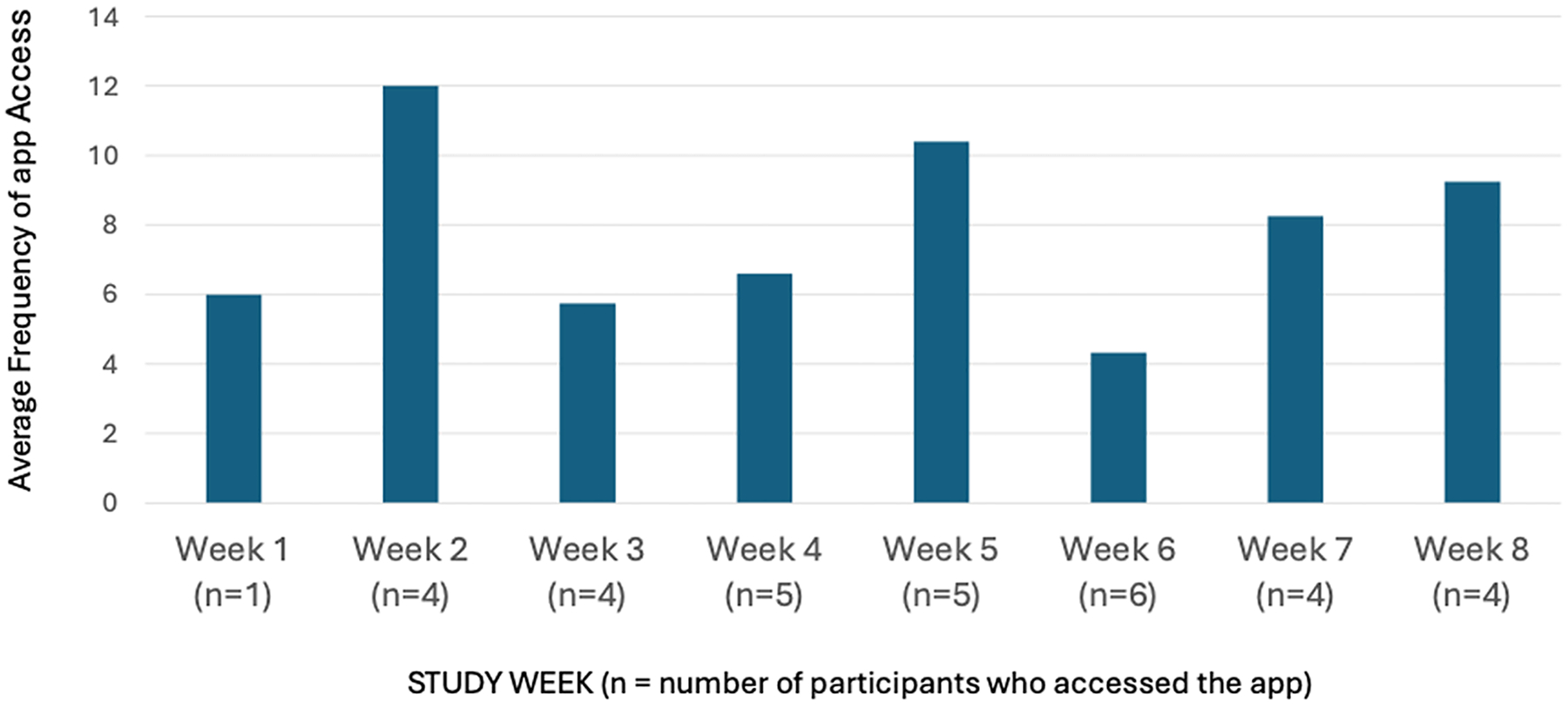
Frequency of MMPH App use.

We invited participants to access the MMPH App daily and use it to support their daily mindfulness practice for at least 20 min/day from the first day of Week #1 through the last day of Week #8. Eight out of 15 participants (53 %) used the App during the intervention period, 6 out of 15 (40 %) used the App for >2 weeks, and 5 out of 15 (33 %) used the App beyond the 8th week of the study period. The average number of times they used the App per week was 5. Fig. 1 represents the average frequency of App use among those who accessed the App. The range of time spent by App users is displayed in Table 5, with the shortest time of use at 0.3 min and the longest at 918.5 min. The frequency at which participants used the App ranged from 1 time to 25 times per week.

After we provided detailed instructions for downloading and accessing the MMPH App, all participants (*n* = 15) logged in to the Daily Check-in function and entered their daily symptom severity ratings for dyspnea, anxiety, pain, and fatigue. The entry frequency ranged from 1 to 58, with an average of 25.53 entries over the 8-week study period.

### Qualitative results

#### Participants’ reflections of MMPH app use

Participants were asked to enter daily reflections into the Reflections section of the MMPH App to document their mindfulness journey throughout the eight-week study period. During the Zoom #1 meeting, we provided verbal instructions, encouraging participants to record any thoughts or feelings that may arise, specifically related to their daily activities. We intended this activity to support and facilitate their mindfulness practice, enabling them to be aware of present-moment thoughts, emotions, and bodily sensations.

Ten out of 15 participants (67 %) entered reflections on their mindfulness journey in the App. The number of reflections was highest in the first week of the intervention (*n* = 23), then declined in the subsequent weeks, increased again until the sixth week (3 records). However, there was a slight increase in the final week, with five entries recorded.

The content analysis reveals four most salient themes, including Perceived Benefits of Meditation (*n* = 20), Daily Life Practice and Activity (*n* = 29), Awareness and Acceptance (*n* = 19), and PH Symptom Improvement (*n* = 11).

##### Theme 1: Perceived Benefits of Meditation

Participants reported changes in their physical bodies and emotions after using one of the app’s exercises, including muscle relaxation, reduced pain, improved breathing quality, increased ability to focus, a sense of calm and relaxation, stress reduction, and lowered anxiety. They reflected on the immediate benefits of these exercises, which affect them both physically and mentally. The most frequently mentioned to be favorable and desirable were exercise modules that explained gentle body movements, mindful breathing, and body awareness meditation. Some participants suggested adding soft background music to enhance the relaxation within the modules.

“I find that the meditations help me to focus on my body and my breath more. I think that I breathe more deeply when I am mindful of what I am doing and feel better.”

“I also have noticed that when I am being mindful of the areas of my body that I feel pain, that I feel the depth of that pain, and when my mind is focused on another part of my body that the pain from the other area doesn’t feel as intense or I don’t even notice it.”

“The movement of my fingertips touching and moving across my cheeks and jawline felt soothing and relaxing.”

“I feel peace and relax after the meditation session.”

##### Theme 2: Awareness and Acceptance

Participants frequently expressed a deep understanding of the mindfulness concepts of positive thinking, gratitude, and increased awareness of acknowledging positive aspects of their lives and their PH condition. Participants mentioned that focusing on positive things gives them more confidence in their bodies and in what they can do. Accepting things brings a sense of peace and tranquility. They learn to be more aware of their feelings and thoughts, which facilitates their ability to accept and live with their bodies.

“Meditation seems to be very calming for me. It makes it very easy for me to put aside worries and stresses and feel calm and in the moment. It also helps me to be more aware of my body and very appreciative of all my body does for me and does well.”

“Mindfulness is teaching me about my thoughts, my emotions, my physical part of me, the way I cope, how I relate to others, what I feel like I can share, etc. It really has opened my eyes and has shown me some things that I need to work on changing.”

“When I am being more mindful things come to me that don’t normally come to me, I appreciate things more, I pay more attention to details and not take anything for granted.”

##### Theme 3: Daily Life Practice and Activity

Participants used the App’s Reflection feature to track their daily life activities, practice plans, recent events, and current moods. These reflections are general descriptions that indicate their ability to notice thoughts and feelings and connect them to what is happening in their daily life. These entries reflect their desire to document their lives and thoughts, and the content encompasses a wide range of topics.

“Today was a very stressful day. In fact, it’s been a very stressful week, and it’s only Tuesday!”

“Feeling good. I have been using the skills, but was unable to get to the phone as it was left in Portland at my granddaughter’s.”

“Rough day today. Anxious about the pending biopsy and life in general. Going to do a body scan practice and some tapping.”

“I have even found myself talking negatively to myself, saying things to myself. Feelings of being out of control arise and therefore cause me to feel angry with myself for allowing it to happen.”

Some participants expressed intention for ongoing use of the practice as indicated by *“I’m going to do breathing and gentle exercise today before I go to bed”*, which showed how much they accepted the practices learned from the app.

##### Theme 4: Management of PH Symptoms

Two participants used this feature to record their feelings about the PH experience and dyspnea symptoms. They noticed that the snowy weather had started and subsequently became aware of its influences on PH management (increased discomfort wearing nasal cannula oxygen) and on other health conditions (worsened joint pain). They mentioned that the colder weather made breathing uncomfortable, and being able to breathe freely became a luxury. With this awareness, they took steps to avoid any potential negative outcome of their changing environment. These proactive actions include keeping their airways warm and ensuring they have enough oxygen supply before going outside. They also recognize the consequences of PH on their psyche (heightened anxiety) and daily activities (limited energy level).

“I noticed that I was getting frustrated with it all. I was having to do so much extra just because the weather had changed. I found myself and my thoughts wandering every which way.”

“Focusing on my breathing, how my hands and feet are feeling due to my Raynaud’s Phenomenon. Lately, I have been staying indoors as much as I can. I had to go get my prescription today, and the temperature was only 22°F. Mindfulness this time of year has so many facets to consider.”

“I am finding with the colder weather that I am experiencing more pain in my joints, particularly my feet.”

“This is when this disease makes you feel so alone. It is hard to make people understand that breathing in colder weather is so much more different for me than it is for them.”

## Discussion

A detailed analysis of participants’ actual engagement with the MMPH program, combined with their qualitative narratives about its use, provides critical insights into the intervention’s acceptability and feasibility among PH participants. Engagement patterns offer objective indicators of how participants interacted with the program (e.g., frequency, duration, and consistency of use). At the same time, qualitative accounts capture participants’ subjective experiences, including perceived relevance, ease of use, and barriers or facilitators to participation. Together, these complementary perspectives deepen our understanding of participants’ willingness to engage with the program, the perceived benefits and challenges associated with its use, and the factors influencing sustained participation over time. Such insights are vital not only for determining the immediate feasibility of the MMPH in supporting participant engagement but also for supporting future examination of its long-term viability, scalability, and potential integration into routine clinical care.

Our findings align with previous research on virtual, multimodal mindfulness-based interventions. For example, a six-week social media–based psycho-behavioral program (MCARE) for patients with acute coronary syndrome achieved an overall completion rate of 76 % (ranging from 68.5 % to 100 % among those attending more than five sessions).^41^ In comparison, our eight-week program reached a 53.3 % completion rate (more than six sessions), which is lower than the rate reported by Zou and colleagues, whose intervention included one face-to-face session followed by five weekly social media–delivered sessions. Similar to Zou’s study, our participants did not consistently meet the recommended frequency or duration of home meditation practice, suggesting a decline in engagement during our longer 16-week intervention, particularly in Group 2. Notably, Zou’s adherence data relied on participants’ self-reported practice, whereas our study captured adherence objectively through app-recorded data.

While the attrition rate was not an issue in our pilot study design, prior studies using MBIs have encountered varying degrees of participant dropout, which appears to be related to researcher-participant communication and interactions, as well as the delivery method of the MBIs. For example, an 8-week culturally adapted online mindfulness training course (MTC) for college students reported a 60 % attrition rate (32/80 completers).^42^ A possible explanation for the high attrition rate in Sarfraz’s study is the absence of remuneration for students’ participation, as the most salient reason for dropout is the lack of time.^42^ Studies that employed strictly online and web-based delivery appear to struggle in maintaining participants throughout the studies, with rates of 74 %,^43^ and 42 %.^44^ An in-person 4-week mindfulness course for women during pregnancy and early motherhood reported a 15 % attrition rate among 48 participants randomized to the intervention group.^45^ While each session lasted about 2.5 h, the face-to-face delivery format was beneficial for this population, given the supportive benefits of group engagement. The MMPH program was designed to strike a balance between group engagement and independent practice, as well as synchronous virtual and asynchronous video-led practice.

Participants’ perspectives on their engagement in these MBIs, as mentioned above, were generally positive, based on SUS scores of 81.47 (±10.21), an attrition rate of 6.25 %, and a completion rate of 80 % among participants who completed > 50 % of the required content. The SUS score in our study was noted as high, indicating strong acceptability of the intervention. Our result is comparable to those reported in previous studies. Ninety-two percent of participants in Wang’s study reported a satisfaction score greater than 8 on the Course Satisfaction Questionnaire (1–10), indicating high satisfaction with the course.^45^ Zou et al. (2024) assessed acceptability using a self-developed dichotomous questionnaire (positive ratings greater than 80 % are considered acceptable), which reported acceptability rates ranging from 93 % to 100 % among participants.^41^ Our acceptability metric was obtained using a validated instrument, the SUS, which captured multiple dimensions of engagement in the MMPH program. Furthermore, our ability to capture their perspectives within the app (just-in-time) of their mindfulness practice 24/7 was invaluable, rather than relying on long-term recall, which can be a barrier among patients with PH. Possible reasons for this strong acceptability include the ease of using the technology, the variety of engagement strategies, or the daily reflection feature embedded in the app, which may have enhanced the user experience.

Our qualitative results corroborate the objective acceptability metric obtained, as indicated by the themes that emerged. They perceived that the MMPH program was beneficial for managing PH symptoms by teaching mindfulness, fostering awareness and acceptance, and integrating what they learned into their daily practice. They appreciated the simple practice of focusing on the sensation of their body parts, such as breathing, to help alleviate day-to-day anxiety and keep them calm.” They notice how they feel after each meditation session, and can connect this positive sensation to the practice. They also recognize that this was the beginning of their mindfulness journey, which requires regular, ongoing practice to become part of their everyday lives.

Participants also offered suggestions that provided valuable insight into opportunities to further refine the MMPH intervention. For example, some participants preferred specific mindfulness activities and frequently rewatched portions of the 60-minute videos. They noted that navigating these long recordings was cumbersome and time-consuming, and recommended dividing each video into four shorter subsections. Participants also reported challenges due to the lack of an Android version of the MMPH app, which prevented them from accessing audio practices directly on their phones and required them to use a web page instead. Additionally, they expressed a need for easier access to prior reflection diary entries to support ongoing review of personal notes and learning. This feedback is invaluable and will guide future enhancements to the program.

### Study limitations

Several limitations must be acknowledged in this study. *First*, the small sample size of our study limits the generalizability of the results. *Second*, the measurement of app use duration may be inaccurate because Android app users must manually turn off app audio, which can result in extended play without actual participation. *Third*, the frequency of participants’ reflection entries declined over time, suggesting an incomplete assessment of participants’ perspectives on app use. Hence, other possible factors may explain this behavior beyond our ability to capture (e.g., lost interest over time due to limited face-to-face communication with the Interventionist/ other participants, as seen in Zoom #1 and Zoom #4).

Regarding video viewing, most participants watched the videos as prescribed; however, their engagement decreased over time. Of 15 participants, 13 accessed the video at least 50 % of the time (3 of 6 videos), and 12 had an average viewing time of 70 % or more. While the content delivery and practice are not perfect, they are considered acceptable by conventional standards of 75 % session attendance (six out of eight sessions). Participants’ decline in video engagement was likely related to the length of the recording, as each session consisted of a single 60-minute presentation. This interpretation was supported by feedback gathered at the end of the program. Although each video contained four distinct practice segments, participants found it difficult to navigate within the full-length recording and recommended dividing each video into four shorter modules of 10–15 min for easier access and repeated viewing. A secondary explanation for the decline in engagement may be the reduced novelty of the practices after the first four weeks, as participants had already become familiar with the content. However, most participants indicated that they liked the visual demonstration of movement and positioning for comfort, which appears to reiterate the practice demonstration during the Zoom sessions.

## Conclusions

Our results support the acceptability and feasibility of video-delivered MMPH content at a reasonable level. While the acceptability and feasibility of the Zoom and Video components of the MMPH intervention were reasonable, participants’ app use was deemed suboptimal. Specifically, the videos could be reorganized and presented in shorter sections rather than 60-minute segments. It is important to note that the crux of the MMPH program component is embedded in the Zoom and video delivery, and the app used is meant to support independent home practice. The results indicated that traditional MBI delivery using these methods is well received by PH participants, but MBI delivery via app requires further enhancements and modifications (technical issues) to better serve their specific needs and demographic characteristics (participants’ preferences). Therefore, it is necessary to allocate resources to modify and enhance the MMPH app to address the issues identified in this study. Relevant improvements and further testing of the MMPH intervention will facilitate its use to empower patients with PH in self-managing PH symptoms and disease, thereby improving health-related quality of life.

## Figures and Tables

**Table 1:** MMPH acceptability and feasibility definitions and metrics.

	MMPH Acceptability		MMPH Feasibility		
**Definitions**	Did participants complete MMPH intervention according to the study protocol?	What were participants perceptions about the MMPH Intervention?	Is MMPH study feasible to be conducted among adults with PH?	Is MMPH intervention feasible to be delivered among adults with PH?	Is MMPH intervention feasible to be completed among adults with PH?
**Metrics**	Adherence Rate Attendance Rate - Zoom Attendance Rate - Video Engagement Rate - App Frequency of App Access Duration of App Access (Total during Study) Duration of App Access (Total per Week) Duration of App Access (Average per Week) SUS-Modified	Qualitative Thematic Analysis	Recruitment & Enrollment Rate Recruitment Time Refusal Rate Acceptance Rate Attrition Rate	Description of Intervention Protocol	Qualitative Thematic Analysis

Note: MMPH, Mindfulness Meditation for Pulmonary Hypertension; PH, Pulmonary Hypertension; SUS, System Usability Scale.

**Table 2:** MMPH program content.

Week & Delivery Method	Week 1 - Group Zoom	Week 2 - Video	Week 3 - Video	Week 4 - Group Zoom	Week 5 - Video	Week 6 - Video	Week 7 - Video	Week 8 - Video
Content	*We Are All Beginners* - Introduction to Mindfulness	*Our* PH *Experiences* - PH symptoms	*Stress & Strength* - Shifting your Perspective	*Mindfulness in Practice* - Midpoint Check-in	*Express Yourself* - Mindful Communication	*Health & Wellness* - Healthy Behaviors	*Resiliency* - Inner & Outer Resources	*Way of Life* - Ongoing Mindfulness Practice
Supplemental App modules Practice	BREATHE-Sit-5-POSE-Sit-5-min, BAM-Sit-5-min, BAM-Back-5-min	BREATHE-Sit-5-POSE-Sit-5-min, BAM-Sit-5-min, BAM-Back-5-min	BREATHE-Sit-10-GBM-UE-10-min, POSE-Sit-10-min, POSE-Back-10-min, BAM-Sit-10-min, BAM-Back-10-min	BREATHE-Sit-10-GBM-UE-10-min, POSE-Sit-10-min, POSE-Back-10-min, BAM-Sit-10-min, BAM-Back-10-min	BREATHE-Sit-10-GBM-UE-10-min, POSE-Sit-10-min, POSE-Back-10-min, BAM-Sit-10-min, BAM-Back-10-min	BREATHE-Sit-20-GBM-LE-10-min, POSE-Side-10-min, BAM-Sit-20-min, BAM-Back-20-min	BREATHE-Sit-20-GBM-LE-10-min, POSE-Side-10-min, BAM-Sit-20-min, BAM-Back-20-min	BREATHE-Sit-20-GBM-LE-10-min, POSE-Side-10-min, BAM-Sit-20-min, BAM-Back-20-min

*Note:* **MMPH**, Mindfulness Meditation for Pulmonary Hypertension; **PH**, Pulmonary Hypertension; **BREATHE-Sit**, Mindful breathing while seated; **POSE–Sit**, Mindful sitting pose; **BAM-Sit,** Body-awareness meditation while seated; **BAM-Back,** Body-awareness meditation while supine; **GBM-UE**, Gentle-body movement of upper extremities; **POSE–Back,** Mindful laying supine pose; **GBM-LE**, Gentle-body Movement of lower extremities; POSE–Side, Mindful side-lying pose.

**Table 3:** Demographic and clinical characteristics of participants.

	Group 1	Group 2	p-value
**Age (years)**	52.5 ± 14.08	46.2 ± 7.98	*0.454
**Years Living with PH**	9.14 ± 10.41	21.0 ± 22.11	*0.341
**Gender**			**1.000
Male	1	0	
Female	8	6	
**TakingPH medication?**			**1.000
No	1	0	
Yes	8	6	
**Taking anti-anxiety medication?**			**0.622
No	6	3	
Yes	3	3	
**Taking anti-depressant medication?**			**0.3287
No	7	3	
Yes	2	3	
**Have Severe Depression?**			**1.000
No	7	5	
Yes	2	1	
**Employment Status**			**1.000
Full-time	2	1	
Part-time	1	1	
Unemployed	1	0	
Not working/ disabled	2	2	
Retired	3	2	
**Education**			**0.622
High School Completed	3	3	
College Completed	6	3	

**Note:** pH, Pulmonary Hypertension;

* Wilcoxon rank-sum test for population median;

** Fisher’s exact test.

**Table 4:** MMPH sessions delivery (Panopto video viewing and zoom attendance).

ID #	Week 1	Week 2		Week 3		Week 4	Week 5		Week 6		Week 7		Week 8	
	Zoom (min)	% of Viewing	Video (*f*)	% of Viewing	Video (*f*)	Zoom (min)	% of Viewing	Video (*f*)	% of Viewing	Video (*f*)	% of Viewing	Video (*f*)	% of Viewing	Video (*f*)
1	90.00	87.30 %	3.00	76.12 %	2.00	0.00	21.52 %	1.00	52.38 %	1.00				
2	90.00	16.51 %	1.00			90.00	99.97 %	1.00	13.28 %	1.00	100.00 %	1.00	78.67 %	1.00
3	90.00	*174.36 %	6.00	7.74 %	1.00	90.00	19.26 %	1.00	22.10 %	1.00	20.70 %	1.00	25.87 %	2.00
4	90.00	99.90 %	1.00	*100.36 %	2.00	90.00	35.45 %	2.00	47.22 %	1.00			72.24 %	2.00
5	90.00	*265.3 %	4.00	*183.19 %	4.00	90.00			77.29 %	3.00	*169.11 %	2.00		
6	90.00					90.00					27.42 %	2.00		
7	90.00	45.00 %	2.00	*103.04 %	4.00	90.00	65.19 %	4.00						
8	90.00			61.61 %	2.00	90.00			*100.99 %	2.00	71.70 %	1.00	4.98 %	1.00
9	90.00	9.98 %	2.00			90.00	7.20 %	1.00					*106.88 %	1.00
10	90.00					0.00								
11	90.00			*101.86 %	2.00	90.00	50.40 %	1.00	97.80 %	1.00				
12	90.00	*102.15 %	3.00	*116.48 %	3.00	90.00	100.00 %	3.00	99.97 %	1.00	*100.51 %	2.00	88.77 %	2.00
13	90.00	99.18 %	1.00	99.90 %	1.00	90.00					79.59 %	1.00		
14	90.00	*113.49 %	2.00	*116.95 %	4.00	90.00			22.92 %	1.00	*107.43 %	2.00	9.81 %	1.00
15	90.00					90.00			22.86 %	1.00	*102.29 %	1.00	16.68 %	1.00

***Note***: MMPH, Mindfulness Meditation for Pulmonary Hypertension;

* attendance time (%) above the prescribed duration of 60 min duration.

**Table 5:** Average Duration of MMPH App use for each Study Week.

Study Week	Shortest time of App use (minute)	Longest time of App use (minute)
Week 1 (*n* = 1)	0.1	2.30
Week 2 (*n* = 4)	0.3	918.50
Week 3 (*n* = 4)	0.3	16.40
Week 4 (*n* = 5)	0.8	31.90
Week 5 (*n* = 5)	0.2	982.00
Week 6 (*n* = 6)	0.4	456.00
Week 7 (*n* = 4)	0.2	115.65
Week 8 (*n* = 4)	0.1	103.70

Participants’ Daily Check-in.

## Data Availability

The data that support the findings of this study are available on reasonable request from the corresponding author.

## Abbreviations:

PH: Pulmonary hypertension
WHO: World health organization
GOLD: Global initiative for chronic obstructive lung disease
MBI: Mindfulness-based Intervention
COVID-19: Coronavirus disease of 2019
MBSR: Mindfulness-based stress reduction
COPD: Chronic obstructive pulmonary disease
PAH: Pulmonary arterial hypertension
CHA: Complementary health approaches
UZIT: Urban Zen integrative Therapy
MMPH: Mindfulness meditation for pulmonary hypertension
BCC: Behavior change consortium
SUS: System usability scale
